# Implementation of surgical debriefing programs in large health systems: an exploratory qualitative analysis

**DOI:** 10.1186/s12913-018-3003-3

**Published:** 2018-03-27

**Authors:** Mary E. Brindle, Natalie Henrich, Andrew Foster, Stanley Marks, Michael Rose, Robert Welsh, William Berry

**Affiliations:** 1 Ariadne Labs at Brigham and Women’s Hospital and the Harvard TH Chan School of Public Health, Boston, MA USA; 2 Department of Surgery and Community Health Sciences, University of Calgary, Affiliate Faculty, Ariadne Labs, Alberta Children’s Hospital, 2888 Shaganappi Trail NW, Calgary, AB T2N0Z6 Canada; 3 Department of Anesthesia and Operative Services, Madigan Army Medical Center, Tacoma, WA USA; 4 Memorial Healthcare System, Fort Lauderdale, FL USA; 5 McLeod Health, Florence, SC USA; 6 Department of Surgery, University of South Carolina School of Medicine, Columbia, USA; 7 Beaumont Health-Royal Oak, Royal Oak, MI USA

**Keywords:** Checklist, Debriefing, Team training, Crew resource management, Implementation, Surgery, Quality improvement

## Abstract

**Background:**

The role of the “debrief” to address issues related to patient safety and systematic flaws in care is frequently overlooked. In our study, we interview surgical leaders who have developed successful strategies of debriefing within a comprehensive program of quality improvement.

**Methods:**

Semi-structured interviews of four implementation leaders were performed. The observations, beliefs and strategies of surgical leaders are compared and contrasted. Common themes are identified related to program success and failure. Quality and safety researchers performed, coded and categorized the interviews and coordinated the analysis and interpretation of the results. The authors from the four institutions aided in interpretation and framing of the results.

**Results:**

The debriefing programs evaluated were part of comprehensive quality improvement projects. Seven high-level themes and 24 subthemes were identified from the interviews. Themes related to leadership included early engagement, visible ongoing commitment and enforcement. Success appeared to depend upon meaningful and early debriefing feedback. The culture of safety that promoted success included a commitment to open and fair communication and continuous improvement.

There were many challenges to the success of debriefing programs. The loss of institutional commitment of resources and personnel was the instigating factor behind the collapse of the program at Michigan. Other areas of potential failure included communication issues and loss of early and meaningful feedback.

**Conclusions:**

Leaders of four surgical systems with strong debriefing programs report success using debriefing to improve system performance. These findings are consistent with previously published studies. Success requires commitment of resources, and leadership engagement. The greatest gains may be best achieved by programs that provide meaningful debriefing feedback in an atmosphere dedicated to open communication.

## Background

Since the publication of “To Err is Human” in 1999, medical error has been acknowledged as a major contributor to the burden of illness in the United States (US) [1]. Preventable complications in surgery contribute significantly to this burden. Over the last 17 years, the quality and safety of surgery in the United States has been addressed through several interventions. One prominent tool developed to improve patient safety is the World Health Organization (WHO) surgical safety checklist [2]. The WHO checklist is a communication tool that involves participation of the surgical team to review issues of surgical safety at three time points: at a sign-in prior to administration of the anesthetic, at a time-out prior to the incision and at a sign-out or “debrief” at the end of the case. Implementation of the WHO Checklist was associated with dramatically improved outcomes in an international trial published in 2009 [2]. Since the publication of these findings, the checklist has been broadly adopted and incorporated into US hospital accreditation with the expectation that this would lead to national improvement in surgical safety.

Despite a concerted, national effort, the burden of medical error in the US remains troublingly high [3]. Follow-up studies of the WHO surgical safety checklist were less promising than the initial trial, and the mandated use of the surgical checklist failed to move the needle of surgical morbidity and mortality within large health systems such as that in Ontario, Canada [4]. These findings suggest that strategies that reduce surgical morbidity within the controlled settings of a trial may fail in the complicated real world. Success or failure of the checklist in improving outcomes closely relate to meaningful compliance [5]. Despite widespread institutional reports of high compliance, audits of checklist use reveal that most hospitals fail to complete the checklist, with very few institutions completing a true team-based “debrief” or “sign out” at the end of the checklist [6, 7]. The potential role of the “debrief” to address issues related to individual patient safety and systematic flaws in care is almost always overlooked and undervalued [6, 8].

The “debrief” within a three-part surgical safety checklist is alternatively referred to as a “sign-out”. These terms are used interchangeably in most checklists and within this paper. However, the use of the term “debrief” emphasizes additional communication beyond acknowledgement of the tasks performed by the nursing team at the end of the surgical case. This additional communication typically addresses safety, equipment and efficiency issues arising during the case and identifies opportunities for improvement.

In contrast to many hospitals that have struggled with checklist implementation, there are a few notable institutions have used the surgical checklist effectively to improve quality and safety and continue to do so. Many of these successful institutions have specifically focused on the debriefing aspect of the checklist to address systematic issues of safety, efficiency, and communication [9, 10]. In our study, we interview surgical leaders from four centers across the United States who have developed successful strategies of debriefing within a comprehensive program of quality improvement. We examine three institutions that have maintained these programs and one institution where this program has failed.

Through a thematic analysis of these interviews, we explore effective strategies, the role of leadership, barriers and facilitators, as well as contextual factors that present stumbling blocks and opportunities for medical leaders looking to improve system efficiency and strengthen the role of debriefing in their hospital.

## Methods

This study was reviewed by the Harvard Ethics Board and judged to be exempt from ethics review.

### Study design

This study presents a thematic analysis of interviews of surgical leaders at four US institutions. The observations, beliefs and strategies of surgical leaders as reflected in their statements are compared and contrasted. Common themes related to program success and failure are identified. Three quality and safety researchers who are not members of the institutions under study generated the study design, performed, coded and categorized the interviews and coordinated the analysis and interpretation of the results. The authors representing the four institutions aided in interpretation and framing of the results.

### Selection of target hospitals

To identify four institutions, a series of searches of the grey literature through Google search engine, word of mouth, emails and phone calls as well as searches using medical search engines were performed. The aim of these searches was not to be exhaustive but rather to identify representative programs that had developed a debriefing protocol with a strategy of process improvement in mind. One of the four target hospitals (McLeod) was known to the lead authors of this study (MB, WB). This program was also asked to identify any additional programs they were aware of that had adopted a similar strategy for debriefing.

Four centers that are geographically and administratively distinct were identified: Beaumont (Michigan), McLeod (South Carolina), Madigan (Washington), Memorial Health (Florida). The programs in South Carolina, Washington and Florida reported ongoing success while the program at Michigan had initial success but ultimately failed.

### Interviews

Medical and administrative leaders were contacted by both email and telephone at each site and asked if they would be willing to undergo a formal interview by telephone. We identified one key medical leader from each site.

A script for a semi-structured interview was created through an iterative process. An initial script was generated, piloted on quality and safety research colleagues and revised. The script was used as a guide for the interview but was not rigidly followed to allow for spontaneous discussion of elements not addressed within the script.

The areas of assessment that were targeted in the interviews were defined by two members of the research team (MB and WB). These included: motivation/rationale for the debriefing initiative, contextual factors, role of local leaders, implementation process, functionality, evolution/sustainability, buy-in and reinforcement. In addition, the impact of the debriefing strategy on process and safety outcomes, team-work and communication was assessed. Each interview was performed by telephone using a web-based recording program and transcribed.

Transcribed interviews were reviewed by the primary interviewer alongside the original recording to ensure accuracy.

### Analysis

Using NVivo, a software program designed for qualitative analysis, the transcribed interviews were coded according to themes. These themes were identified through two-stage process including deductive themes based in part on the predetermined questions and inductive themes that emerged from the content of the interviews. Each line of transcribed text was reviewed for ideas, concepts and reflections which were categorized by thematic codes. Each new idea or concept was assigned to an existing thematic code or a new code was created. At the end of coding all the interviews, each interview was reviewed again to ensure consistent coding across interviews. Coding was performed by a single reviewer. After coding, code validation was performed by a second qualitative expert who reviewed the content and coding themes for consistency. If discrepancies in coding were determined, discussion among the authors was performed until consensus was achieved. The coded themes were reviewed and broad categories encompassing these themes were created.

Narratives were collected within thematic groupings. Common observations were reviewed and discussed by the research team including the clinician leaders who had been interviewed from each site. The elements that were commonly linked to success were categorized; the challenges encountered by each program were, likewise, categorized.

The program at Beaumont, Michigan, was examined as a separate case study. The threats to the success of the Michigan program that eventually lead to its collapse were identified.

How these threats might be prevented or mitigated were reviewed and are presented within the discussion.

All narratives are presented as verbatim. Any changes that are provided for clarification are presented in square brackets.

## Results

The four implementation leaders interviewed had developed their strategies within four very different health systems.

Madigan is an Army Medical Center in Washington that performs roughly 10,000 surgeries a year and has 220 inpatient beds. Madigan is staffed by 54 surgeons, 12 anesthesiologists and 49 certified registered nurse anesthetists (CRNAs). Dr. Andrew Foster is Chief of Anesthesia at Madigan and was a physician leader who took on a primary role in creating and maintaining the debriefing program at Madigan.

Memorial Health is a large public health system in Southern Florida including five hospitals with a total of 1858 beds and 62 ORs staffed by 545 surgeons, 101 anesthesiologists and 53 anesthesia assistants as well as 112 CRNAs. Dr. Stanley Marks is a surgeon and Chief Medical Officer for Memorial Health, who developed a comprehensive approach to patient quality and safety that has been instituted across Memorial Health.

McLeod Health is a large private health system including seven hospitals in South Carolina. McLeod regional center completes 19,000 cases a year and has 489 beds. The OR is staffed by 125 surgeons, 12 anesthesiologists and 50 CRNAs. Dr. Michael Rose is an anesthesiologist and Vice President of Surgical Services at McLeod Regional Medical Center and has helped lead a state-wide initiative to improve Surgical Safety in South Carolina.

Beaumont is a large public health system including eight hospitals and performing more than 50,000 surgeries every year in Michigan. The system is staffed by 600 surgeons, 84 anesthesiologists and 145 certified registered nurse anesthetists. Dr. Robert Welsh is Vice Chief of Surgical Services at Beaumont Hospital- Royal Oak and developed the program of surgical debriefing for surfacing safety defects at Beaumont Hospital.

In analyzing the interviews of these leaders, seven high-level themes were identified with an additional 24 subthemes that fell within the high-level umbrella themes.

**Fig. 1:**
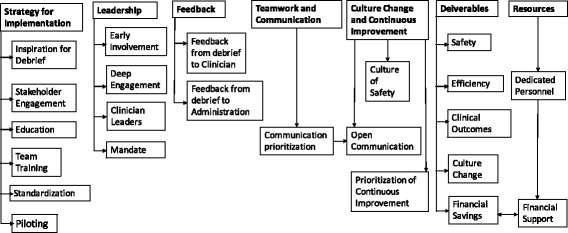
Themes Identified in Interviews

Figure 1 illustrates the broad thematic categories as well as the subcategories.

### Implementation of the debriefing program

The clinician leaders we interviewed all adopted primary leadership roles in initiating programs as well as promoting the importance of the program within the clinical teams and executive leadership. These leaders are passionate, articulate and deeply committed.

#### Team training

Each of the four programs we present used a team training approach as the structural framework on which their programs were built. Different programs used different team training platforms. Two of these programs adopted the formal team training program TeamSTEPPS™ (Memorial Health and Madigan) [11] while two program adapted team training programs from non-surgical specialties. McLeod adapted an airline safety model from FedEx while Beaumont adapted the Keystone ICU team-based quality improvement strategy [12]. Common elements to all these programs included a strong focus on teamwork, communication and clinical leadership all with a goal of improved patient safety. Standardization was important both within single centers as well as between centers operating as part of a single health system.

#### Role of debriefing within a larger program of quality improvement

Although each interviewee reported that debriefing was part of a much larger implementation strategy at their centers, they acknowledged that the debriefing offered a very specific, crucial contribution to quality improvement. For each program, the debrief aims to identify and address flaws in the system and improve patient safety and system function. Education, piloting, stakeholder involvement and feedback are involved at all centers as strategies used to design and implement programs.

#### Operationalization of the debrief

The operationalization of the debrief differed from site to site. The debriefing is generally run by nurses but sometimes this responsibility rests with the surgeon. Participation of all members of the surgical team is an expectation in all programs. The physical form of the debrief itself is not consistent. While McLeod and Michigan use paper systems, Memorial uses an established electronic system and Madigan is working with an electronic system that is being revamped. Many of these systems rely on an administrator who categorizes the issues identified in the debrief, assigns tasks to address the issues and provides feedback regarding the plan and eventual resolution. These programs have all reported tremendous value from their programs in teamwork and communication, satisfaction and measures of efficiency and patient safety [9, 10, 13, 14].

### Factors key to program success: leadership

All four interviewees identified executive leadership as important in both creating and maintaining programs (Table 1). Early leadership participation in establishing and prioritizing the program, ongoing on-site engagement as well as the creation of enforcement measures all represent different ways in which the administrative leadership helped to support programs.

**Table 1:** How Leadership works to Promote a Successful Debriefing Strategy

Theme	Memorial Health, Florida	McLeod, South Carolina	Madigan, Washington	Beaumont, Michigan
Early, Immersive Engagement of Executive Leaders	“I wound up getting all of the executives trained in team training. So people… who had probably never set foot in an operating room were all trained in team training and crew resource management.” “we trained all our senior leaders in the organization…. nobody was excluded.” “[The Crew Resource Management Program is] governed at a pretty high level. It’s governed by the Chief Medical Officer of the healthcare system and the director of crew resource management for the system.” “the army has to be your senior leaders…. the senior leaders have to embrace it and have to continue to train it and live it.”	“Several of our senior leaders were at the IHI… they came back from that meeting with the outlook that the IHI was going to try to attack the issue of improvement in surgery, and so that began the work.”	“we had a couple sentinel events and the executive leadership at our hospital said “you guys need to change something” and the solution they came up with was team STEPS” “The Deputy Commander who really pushed this, is a private pilot, which is really kind of interesting, and so he is very familiar with … CREW resource management and he was really into this” “I think that the big lesson was you know early, getting senior leadership buy-in, really socializing it, and anticipating the kind of push back you’re going to get and addressing those in very detailed manners, you know don’t leave that up to other people’s discretions on how that’s going to go”	“Our CEO… was a pharmacist or a pharmacy tech when he first started out in the world of healthcare, so he had some engagement with personnel and with patients to some degree. He recognized the success of the Keystone ICU project, and so he was willing to endorse the process going into the operating rooms as well. So we really had [support] at the highest level of the hospital.”
Involvement	“The CEO of the hospital makes rounds in their own hospital. The CMO makes rounds, the chief medical officer of each hospital. I expect all my senior-most to make rounds. I don’t need them in their office, I need them out on the floor. And they see me in all of our hospitals.”	“We actually brought our senior leadership group and board members into the operating room, that corridor, to show them what we were finding…. by the time of our annual board retreat that year, some number of months later, we as an organization … said that we were going to attack this issue of safety in surgery with vigour to attempt to get it to zero rate of harm.” “The ability to reach out to senior people, up to including board members and have them engage at that level with staff as a point of emphasis on the importance of the work is part of the secret sauce here” “when you put board members in scrubs in the operating room, observing and talking to staff… that makes a big difference”		
Enforcement	“I believe that leadership, number one, mandates it and stands behind that mandate. If a member of the medical staff doesn’t take the course and cooperate with the policies, they can’t operate here.”	“we [the executive leadership] don’t go down and beat people up for not providing it but on the other hand what we will do is we will regularly reach out to people and say “I didn’t get a debrief from your case yesterday”, and when you do that for 2 or 3 or 4 years, there aren’t very many circulating nurses that will fail to submit the debrief.”	“It was very important to us to have senior leaders there that said ‘this is what we are going to do, and it really isn’t an option not to participate in this’ ” “We have an officer evaluation form…. It’s an opportunity for your supervisor and you to sit down and say what are the goals here? What are the expectations?... The expectation is you will fully participate in the surgical brief and debrief…. If you didn’t do that well, you might actually do poorly on your evaluation which can have adverse consequences for getting a bonus or for getting a promotion, specifically in the military.”	
Clinician Leadership and Engagement	“I brought to bear the notion of my own experience in the operating room…. And I was able to convince the senior executives, the non-physician senior executives that this was an approach that would make our operating rooms and our procedural areas much safer.”	“this culture owns it because [those of us] that generally live one floor above the action really don’t have to drive this. It is substantially driven by the rank in file staff and their immediate managers who are accountable for doing the fixes.” “a lot of the remedies are in the ingenuity and the creativity of the front line staff.” “we do have a very engaged workforce, I would say that in the final mix of this were physician led, evidence based, data driven”	“our very first day we were there, we had the Chief of Surgery, the Chief of Anesthesia go through each room, if there was a problem we addressed it right then and there” “the other thing I think is really important is surgical leadership…. If you don’t get our surgeons to go along with this, it just won’t happen. We have been very fortunate here; both our Chief of Surgery and our Deputy Commander for Surgical Services are huge components to this. I think that, probably more than anything else has made the biggest difference.”	“My experience had been in the ICU, because I’m also a surgical intensivist, was that the communication piece was the critical component.” “in my room when I’m operating, it’s me-the surgeon-[who leads the debrief] and I generally like to see the surgeons doing that… If the surgeon is taking the time to lead this process, there must be some value to it.” “[Nurse leader in quality and safety] definitely had the respect of the OR personnel, she also had the respect of the surgeons too. She was a little unique in that she was able to walk in both worlds comfortably for her and people accepted her from both the surgeon’s perspective but also from the OR personnel.”

*IHI* Institute for Healthcare Improvement

#### Early, aggressive administrative engagement

At each site, the highest levels of administrative leadership played an important role at the outset of implementation. At Madigan and at MacLeod, executive leadership provided the initial impetus for program development, giving clinician leaders an opportunity to create a process with strong administrative support. In Florida, the executive was convinced by local physician leaders and subsequently made the program a priority. In Michigan, there was strong endorsement of a debriefing program that was established by clinician leaders. The leadership commitment required was described by Dr. Stanley Marks: “*the army has to be your senior leaders… the senior leaders have to embrace it and have to continue to train it and live it*”.

#### Executive experience with quality improvement (not necessarily in surgery)

Although some administrators had never set foot in the operating theaters prior to the implementation of these programs of system improvement, many executive leaders had experience in other system-wide quality improvement strategies. Individual experiences ranged from experience in the flight industry, work within the commercial industry as well as direct involvement with medical quality and safety. Dr. Michael Rose described the quality improvement experience of one of their hospital board members:

*“His job had been with [a large national manufacturing company… that had a very high work force injury rate and very high workforce turnover from poor morale; and the last major project that he did with [the company] … was coming in and creating a culture of safety…. I think they got up to something like 1,000 days without an injury before they had their first one which was relatively minor but it was the turnaround on their culture of safety and their injury rates, so he was quite an expert in general execution in the cultural aspects and the kind of seriousness and support that you needed from leadership when you took on an effort like this.”*

#### Ongoing, visible, on-site administrative involvement

The programs that remained successful all reported strong ongoing engagement of leadership. At McLeod and Memorial health, this engagement took the form of executive staff being physically present in the operating rooms and other areas where clinical care takes place. This physical presence performed two functions, 1) keeping the executive staff aware and more directly connected with the issues faced by the front-line staff, and 2) reinforcing to the front-line staff the genuine commitment of the institution to the process. Dr. Michael Rose summarized the importance of this onsite presence; “*the ability to reach out to senior people, up to and including board members and have them engage at that level with staff as a point of emphasis on the importance of the work is part of the ‘secret sauce’ here*”. In Memorial Health Care and McLeod, the ongoing direct involvement of the highest levels of executive staff remained an important part of the program long after the original implementation.

#### Institutional mandates

An institutional mandate has helped to maintain the debriefing programs at Memorial Health and at Madigan. At Memorial Health, participation in team training was directly tied to the privileging of staff while the military structure at Madigan allowed reinforcement to be tied to military promotion and discipline. The leadership at McLeod engaged staff participation through repeated and direct communication between administration and frontline staff. Using an institutional mandate as a method of achieving participation was described by Stanley Marks; “*I believe that leadership, number one, mandates it and stands behind that mandate. If a member of the medical staff doesn’t take the course and cooperate with the policies, they can’t operate here*.” Dr. Andrew Foster described a similar approach at Madigan “*It was very important to us to have senior leaders there that said, ‘this is what we are going to do, and it really isn’t an option not to participate in this*’.”

### Factors key to program success: creating a culture of safety

Developing a culture of safety and using meaningful feedback to generate a pattern of continuous improvement are common themes that arose during the interviews (Table 2). Creating and maintaining a “just culture” dedicated to surgical safety involved a focus on empowering surgical nurses and encouraging open communication.

**Table 2:** Elements of a Successful Debriefing Program: Creating a Culture of Safety

Theme	Memorial Health, Florida	McLeod, South Carolina	Madigan, Washington	Beaumont, Michigan
Timely. Meaningful Clinical Feedback	“if something occurred yesterday in the operating room, in this morning’s team huddle, of that entire division, they’ll talk about whatever that issue was at a debrief that happened the previous day.” “if you don’t feed it back, then it’s not part and parcel of your performance improvement program and then it just becomes a task. And I think, in places where this does not succeed it is merely a task”	“if you’re going to ask people to be observant, to see it, and you’re going to ask them to tell you, to say it, then you have an obligation to fix it” “the surgeon will say “make sure that goes in the debrief” or anesthesia will say “make sure that goes into the debrief” because they want to see that their headline got put into the paper.” “if they have investigated and resolved then they will see the resolution… and then we’ll come back to those people directly after the fact if… we didn’t get an immediate closed loop solution.”	“Some surgeons really buy into it and say “okay this is what happened today, this is what I want to address. Please put this in the debrief, and I really want to get feedback on it”.”	“We said “you will be responsible for taking the defect that has surfaced, resolving it, but also getting feedback to the personnel who were involved when the defect was identified”. We knew that if we didn’t do that the personnel, and more importantly the surgeons, would not endorse this process.” “the orthopedic guys, they are quick to point out problems and they were complimentary of the process saying “if I put it in the debriefing, I know it’s going to get attended to”.”
Feedback to Executive Leadership	“they’ll talk about whatever that issue was at a debrief that happened the previous day. The information is aggregated and fed back at departmental meetings”	“We report out the results now every year and, well, there is a quarterly written report and we generally do an oral report.”		
Focus on Communication	“improved and excellent communication that occurs between all parties in these areas leads to improved safety and that’s their feeling and I think that they feel safer in the organization.”	“The assistants at the table who were… arguably some of the most critical people on the team with respect to errors because of what the instruments that they are handing, specimens that they are managing, drugs that are passing from the non-sterile to the sterile, that as an example is a group of people who felt they had the least amount of respect, the least amount of ability to influence events, but once released and empowered had a huge impact” “[it is] important… to be attentive to the psycho-emotional wellbeing of the workforce… it’s going to be a learning environment, respect and civility is going to be a priority.”	“we wanted to be quiet because we were afraid to speak up and now I think that’s changed, I think people are much more collaborative.” “I have much more open ability to talk to surgeons and other staff. I know a lot of the nurses and techs on a first name basis which I never did before because we encouraged people to use their first names during the surgical brief. And we are starting to understand that if we don’t communicate this way that the patients don’t actually get the best outcome… introducing each other and talking about stuff has made a huge huge difference.”	“the communication piece was the critical component; it was decreasing the hierarchy so you could promote communication.” “I always started with the most junior person in the room, I didn’t want them to not feel comfortable about speaking up about something they saw when somebody else said something different as to what their observation was.” “by promoting communication, I had heard things I never heard before, people were willing to speak to me, they were fearful of speaking to me because of my title”
Continuous Improvement in Patient Safety	“you’ve got to bring them at least to a minimum standard. Number one. And then, number two, you’ve got to continually move the minimum standard to the right side of the equation- so that you narrow your bell curve…. Raising the bar continuously must become an organizational imperative.” “If you’re not constantly reinvigorating the team component, it has a propensity to deteriorate back to siloed activity and poor communication skill.” “the training itself and the rounding need to be used to reinvigorate the process. I think that if you train once and forget it, you will, at best, have a task. But if you reinvigorate that learning so that it’s done every day and becomes part of the culture of the organization” “although I think we’ve done pretty well, I’m totally of the opinion that we have to continue… this is a work in progress... and we will have to continue to stay focused in this area if we expect continued improvement.”	“the reporting cycle times are so short, both the financial ones and the medical ones… that the experimentation and the piloting can happen on short cycles and so we learn from it, and when you learn from it then it gives you the idea of the next thing that you want to do with it, and the next thing, and the next thing.” “prior to beginning this work, about 30% of the people polled in our operating room using the culture of safety survey felt that it was safe to have surgery in our operating room… we saw a huge change in the culture of safety in the operating room and the comfort level that people had: safety mattered, that somebody was listening, somebody would do, somebody would act”	“to be mindful, and that’s what we’re trying to become-a higher reliability organization. That’s one of the things that we tell people is that mindfulness, that session with failure points, that taking the time out of that busy, the business of your life of your clinical practice or whatever have you and stop and really pay attention to what went right here and what went wrong here”	“in surfacing the defects the idea was that if you could identify these identified patterns, hopefully you could prevent their occurrence in subsequent operations.”

#### Empowerment and “Leveling the Playing Field”

Each of the leaders interviewed acknowledged that the traditional hierarchical approach to communication posed a threat to patient safety. Leveling the playing field allowed all members to recognize and report threats to patient safety and improve communication. Dr. Michael Rose described this process as giving voice to “*a group of people who felt they had the least amount of respect, the least amount of ability to influence events, but once released and empowered, had a huge impact*”.

Empowering nurses means providing them with support from the highest levels of administration. Dr. Andrew Foster describes the structure of support at Madigan in Washington: “*… we have empowered our nurses and techs to say ‘hey, we’re not bringing this patient back until we’ve talked; because number one I’ve been empowered to speak up if I see a safety issue; and number two I’ll go to my leadership, I’ll go to your leadership as a matter of fact.’”*

Creating an atmosphere of open communication was often described as a responsibility that rested with the surgeon; who would invite input from all members on things that could have been done better. To emphasize that everyone’s voice was valued, the surgeon would first ask those who were traditionally less likely to speak up. Dr. Robert Welsh is a clinician leader who exemplifies this approach: “*Even if it was a medical student in the room, I would ask the medical student [to speak first in the debrief] and half the time they had no clue what we had done; but sometimes they might have seen something; and then it was on to the next level trainee, moving all the way up in to the most experienced people; and I usually went last*.”

#### Feedback on debriefing issues to stakeholders

Feedback on the issues raised in the debrief was commonly regarded by the clinical leaders as the cornerstone to successful debriefing. Providing caregivers with early and meaningful feedback on identified problems gave the participants the satisfaction of seeing their problems recognized and prioritized, allowed repetitive issues to be addressed and gave the participants faith that the debrief achieved a purpose. Dr. Robert Welsh reported that even the surgeons who were resistant, became fans of the process: “*they were complimentary of the process saying, ‘if I put it in the debriefing, I know it’s going to get attended to*’”. Feedback provided to the executive also provided an additional way in which leadership could be further engaged in process improvement.

Focusing a system on continual improvement is a strong part of creating a culture that focuses on surgical safety. The process is described by Dr. Stanley Marks: “*you’ve got to continually move the minimum standard to the right side of the equation so that you narrow your bell curve…. Raising the bar continuously must become an organizational imperative*”.

### Challenges to the debriefing program

Each of the leaders interviewed spoke about challenges that they had encountered in the process of implementing the debriefing program and challenges that pose a constant threat to ongoing performance (Table 3).

**Table 3:** The Challenges Encountered in Developing and Maintaining a Debriefing Program

Theme	Examples
The Loss of Leadership Support	“the new CEO… came in probably around October of 2008 and just got blindsided by what happened in November of 2008 so his emphasis now was the bottom line… he’s trying to figure out “how do I keep this boat afloat?” and so if it wasn’t direct patient care they weren’t going to fill those positions” MI
Communication and Cultural Challenges	“I think sometimes surgeons speak one language and nurses speak another and internists speak another and if you magnify that throughout the healthcare enterprise you know maybe there’s 50 languages being spoken in a hospital, all of which is presumed to be English.” FL “I think we’re really going to have to teach people to communicate especially health care providers at all levels. Because it’s become a huge challenge.” FL “I don’t think we communicate well with each other. I think that there are a lot of hand-offs in health care. And each one of those handoffs is a potential red flag.” FL “We had issues of respect and civility in the environment that created some vulnerability and increased the risk because people might be afraid to speak or raise their hand.” SC “when a person feels that they don’t have voice or their voice is suffocated, I think they fundamentally lack confidence and disengage. Some will just check out, but some people will actively disengage and broadcast how the place doesn’t care about anything that happens, never does anything.” SC “it was just supposed to be a verbal process, and I can tell you that the managers, it’s very interesting how people don’t want to talk to each other and for some reason the nurse managers were reluctant to do this, and whether they felt it was just another imposition on their time” MI “we had an education part that would go out to the personnel in the operating rooms. We did not do this with the surgeons, we kind of knew we would have a lot of push back from the surgeons and I thought we’re not going to fight this fight with them” MI
Lack of early, meaningful feedback	“pretty soon, no one is collecting the data anymore, and there’s no question that if you don’t get that data and then give feedback to people, they’ll just stop giving it, they don’t see any value in it anymore.” MI “if you’re in a workforce and you’re doing critical, complicated, potentially deadly stuff and you see adverse events happening and you experience them two weeks ago and the same thing happened six months ago and you predict it’s going to happen again two months from now, you can see how you begin over time to resent it, feel cynical, feel disempowered, and that you can’t make a difference.” SC “Now what they will tell you, though, is “well, I put this stuff in and it doesn’t go anywhere” or “I never see that things change” and that’s something that we all struggle with” WA “the debrief, as I said, is a little bit more out in the cloud, abstract, and we haven’t made a system yet where the feedback is more immediate.” WA
Lack of perceived “value”	“When you go to them and say “look every surgical site infection costs the hospital 11,000 dollars” or whatever number you want to use, they say “yeah, okay that’s nice but did it make any money for us?” and they just couldn’t grasp this.” MI “I think that there’s value that is hard to parse and be able to show as being more cost effective… it would be hard to do.” WA
“Task” mentality	“we had a wrong sided hip surgery done where everybody was like, “oh yeah we did the briefing, we did the briefing”; and what they did was perfunctory. Nobody was engaged, and so the recognition was that we needed a script that called out to people to actually answer a question.” MI “So raising the bar continuously must become an organizational imperative. If you don’t do that, you’ll stagnate.” FL “there’s the drudgery of “do I have to produce this piece of paperwork?” but then yes there’s drudgery that you have to produce this paperwork and it’s another piece of paperwork. If that were the only thing I think that it would be one of those things that we’d have to continuously manage compliance.” SC “for me, it’s the technological hurdles that you know we are in the military, so we want to make sure that our permission systems are highly secure but that rubs up against our desire to also have ease of access and usability.” WA
Loss of Resources	“people were increasingly asked to take on more responsibilities and in fact beyond the ability to do all of them well.” MI “when November 2008 came the belt-tightening was significant and so now it was quite common that when somebody left their position to go to another position, their position went unfilled. So we no longer had the glue person.” MI “So we end up having a replacement for M_ but this person now gets asked to do an audit for this and for that, and a variety of things, so she gets pulled in a multitude of different directions and then she gets another position as well, and the hospital doesn’t see fit to make the replacement” MI

MI- Michigan; WA-Washington; SC- South Carolina; FL- Florida

The loss of leadership support and resources were concrete threats to the ongoing effectiveness of these programs. In Michigan, Dr. Welsh describes the change in leadership as coinciding with a change in institutional priorities: *“the new CEO… came in probably around October of 2008 and just got blindsided by [the economic downturn] in November of 2008 so his emphasis now was the bottom line… he’s trying to figure out ‘how do I keep this boat afloat?’”*

In addition, breakdowns in communication lead to the reluctance of disempowered team-members to speak up and lead team members to disengage with the process.

Dr. Stanley Marks provided an illustration of the wide-spread issues with communication in health care systems: *“I think sometimes surgeons speak one language and nurses speak another and internists speak another and if you magnify that throughout the healthcare enterprise you know maybe there’s 50 languages being spoken in a hospital, all of which is presumed to be English.”*

Dr. Michael Rose described the feelings of disillusionment that lead to disengagement in a system that fails to achieve an atmosphere of open communication: *“when a person feels that they don’t have voice or their voice is suffocated, I think they fundamentally lack confidence and disengage. Some will just check out, but some people will actively disengage and broadcast how the place doesn’t care about anything that happens, never does anything.”*

Providing early meaningful feedback was frequently identified as a key to team members valuing the checklist. When feedback was no longer reliably provided, leaders recognized that this represented a great threat to meaningful participation with degeneration of the debriefing and the entire checklist into a “task” or complete abandonment.

Dr. Michael Rose describes the dangers of the lack of meaningful feedback: *“if you’re in a workforce and you’re doing critical, complicated, potentially deadly stuff and you see adverse events happening and you experience them two weeks ago and the same thing happened six months ago and you predict it’s going to happen again two months from now, you can see how you begin over time to resent it, feel cynical, feel disempowered, and that you can’t make a difference.”*

### Beaumont hospital, Michigan: Why did it fail?

**Fig. 2:**
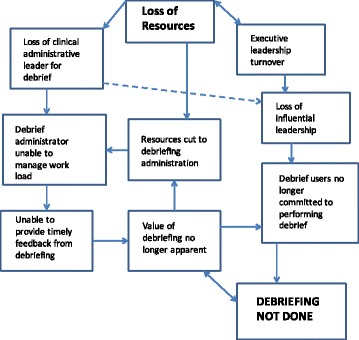
System Collapse at Beaumont, Michigan

Beaumont hospital faced many of the same challenges that other programs faced. Unlike these programs, the system eventually collapsed. An examination of the processes that eventually led to the program’s failure illustrates the fragility of these systems (Fig. 2).

Dr. Robert Welsh identified the financial crash of 2008 as being the catalyst for the program’s collapse. This occurred at the same time as a change in executive leadership. Although executive leadership had supported the program, their involvement was at more of an arms-length than the other programs we have examined and this leadership change further distanced the leadership from the debriefing program. The economic advantages of a debriefing program were not easy illustrated and operated based on costs saved or avoided rather than revenue generated. Robert Welsh describes how the economic impact of 2008 affected the administration’s views on revenue generation and cost-savings:

*“They don’t see that costs avoided are worth considering as much as…“how did I negotiate a contract or save money?” or “what did I do to increase the income?”… the averted cost was just not something that they were willing to recognize. When you go to them and say ‘look every surgical site infection costs the hospital 11,000 dollars’ or whatever number you want to use, they say ‘yeah, okay that’s nice but did it make any money for us?’ and they just couldn’t grasp this.”*

The debriefing program was not seen as a priority to the administration in a situation of scarcity and the key position of the quality and safety nurse was gradually phased out. The nurse in this position facilitated assigning responsibility for dealing with debriefing issues and providing feedback. Once this process was no longer performed adequately, those using the checklist no longer saw the value of the process and stopped participating in this. This loss of feedback was regarded by Dr. Welsh as the key aspect that led the program to collapse: “*…pretty soon, no one is collecting the data anymore, and there’s no question that if you don’t get that data and then give feedback to people, they’ll just stop giving it; they don’t see any value in it anymore.”*

With the change in the executive leadership, the voiced institutional support of the program was also missing and the clinician leaders of the debriefing program no longer had the support of the administration and the culture of safety was no longer a prevalent force.

## Discussion

Improving surgical safety through debriefing is a complex endeavor. Although the role of debriefing is acknowledged as an important part of surgical safety, debriefing is rarely performed in institutional audits of checklist compliance [5, 7]. We have examined four programs that have effectively instituted debriefing systems to improve patient safety and the overall effectiveness of the health system. The leaders of these systems have commonly identified strong executive leadership involvement, meaningful and early feedback and a culture of safety and continuous improvement as keys to their success. Each program recognizes ongoing threats to the success of their programs. These threats are those of resources (financial and personnel), the loss of meaningful and early feedback, and the perseverance of traditional hierarchies resulting in the failure to achieve fair and open communication. In the case of Michigan’s debriefing program, a loss of resources was the catalyst to the eventual collapse of the debriefing program primarily when meaningful feedback was no longer provided to the surgical teams.

Debriefing is well-established in many high-acuity disciplines where it contributes to a culture of communication, reflection and continuous improvement. The American Heart Association strongly promotes the use of debriefing to improve the running of codes [15]. Studies of debriefing after codes demonstrate improved team communication as well as improved performance measures and clinical outcomes. [16–19]. Similar results are seen when debriefing is applied to trauma care [17, 20]. The four interviewees in our study all pointed to numerous ways in which debriefing in surgery contributed to improved clinical care, patient safety, communication, attitudes and efficiency at their institutions. Although patient safety is the priority for each of these programs, the debriefing also offered an opportunity to improve performance measures related to process- expanding the potential value of the program and acting, in part, as a Trojan Horse to introduce important safety alongside those measures that improve the business-side of surgical systems. Published results from Madigan, Washington specifically show improvement in operating room efficiency [10] while the program at Beaumont found numerous communication and equipment issues that improved with the use of debriefing [9]. In institutional publications and presentations, the Memorial Health System in Florida reported improvements in communication, measures of satisfaction, teamwork and safety culture [14] while McLeod Health reported improvement in surgical mortality, a decrease in patient safety events and an improvement in measures of satisfaction and attitudes surrounding safety [13].

There is no simple answer as to why debriefing is not adopted routinely after surgical procedures. Qualitative studies through surveys and interviews suggest multiple reasons including logistical challenges and a perceived lack of value [6, 8]. The logistical challenges relate to the different times at which team members’ roles conclude in surgical procedures and the competing requirements of circulating nurses who are frequently tasked with running the debriefing. The perception that debriefing lacks value relates to the fact that some elements are redundant, other elements may not be directly applicable to the care of the patient and issues arising in the debrief are not addressed or valued by the institution [8].

The lack of a strong culture of surgical safety likely contributes to the devaluing of the debriefing both by the institution and by the team members who directly participate in the care of the patient.

The interviewees from each of the four sites recognized that an immediate tangible value of the checklist would best ensure that the surgical team would participate in debriefing. Often this meant addressing concerns that impacted the teams’ ability to perform its job efficiently in addition to other issues related to improving performance. The fact that issues of equipment and process could be corrected and the surgical team could experience the immediate benefits of participation, gave them a greater commitment to the process.

To improve the culture of surgical debriefing and create a meaningful debriefing process, interviewees acknowledged that the institutional approach should be broad-sweeping, targeting all levels of care and administration with a strong focus on empowerment of nurses and those members of the surgical team who have traditionally not had their voices heard. To create a system in which nurses are empowered, the focus needs to switch from individual performance to team success. In most situations, this requires an institutional culture change. Changing the culture of an institution requires a comprehensive strategy in which team training may play a critical role.

When quality and safety tools are applied without an institutional strategy for ongoing implementation, they are likely to fail. This can be seen in studies of surgical safety checklist that do not specifically involve implementation strategies [4, 21]. When the checklist is introduced as a tool to facilitate communication within a broader institutional strategy, the impact can be tremendous [22]. Programs that succeed in developing debriefing programs to improve surgical safety must remain committed to ongoing improvement and a commitment to communication.

There are many threats to the ongoing success. At Beaumont Hospital in Michigan, the loss of resources was devastating to the debriefing program as key positions were defunded. It is possible that if the executive administration valued the program more either economically or within a stronger institution-wide culture of safety, economic scarcity might not have had the same impact. Stanley Marks at Memorial Health in Florida reflected that the value of the program is so engrained within the institutional culture that it is not likely to be impacted by scarcity. But maintaining the institutional culture takes work. Even in programs like Memorial Health, Stanley Marks refers to the constant threat of degeneration into a “task” mentality.

## Conclusions

The value of the debriefing systems established by four surgical centers in diverse settings across the United States is tremendous. Even in Beaumont, where the system failed, there remains a strong commitment by clinician leaders to re-establish the program. The fragility of these systems exposed by the collapse at Beaumont underlines the importance of strong executive involvement, a reliable system of meaningful feedback and prioritization of communication within a system designed to constantly improve.

## Abbreviations

US: United States
WHO: World Health Organization
